# Assessing the impact of community health education programs on preventing non-communicable diseases in rural areas

**DOI:** 10.1186/s12889-025-22620-8

**Published:** 2025-11-27

**Authors:** Collince Odiwuor Ogolla, Bernard Guyah, Apollo O. Maima

**Affiliations:** 1 School of Public Health & Community Development, Maseno University, P.O. Box 3275-40100, Maseno, Kenya; 2 School of Pharmacy, Maseno University, P.O. Box 3275-40100, Maseno, Kenya

**Keywords:** Health education, Non-communicable diseases

## Abstract

**Introduction:**

Non-communicable diseases (NCDs), such as cardiovascular diseases, diabetes, and obesity, are among the leading causes of mortality worldwide, particularly in rural areas where there may be limited access to healthcare services and health education. The increasing prevalence of these diseases in rural communities underscores the importance of effective prevention strategies, such as community health education programs.

**Objective:**

This study aimed to assess the role of community health education programs in improving knowledge, behaviors, and health indicators related to the prevention of NCDs in rural populations.

**Methods:**

Data were collected from 108 participants in rural areas to evaluate the impact of a community health education program. Surveys focused on health knowledge, lifestyle behaviors (physical activity, diet, tobacco and alcohol use), and health indicators (blood pressure, BMI, and fasting blood glucose). Data were collected at two points: before the community health education program was conducted and after an 8-week period.

**Results:**

The study found notable improvements in health knowledge, lifestyle behaviors, and reductions in health indicators associated with NCD risk factors, such as hypertension, obesity, and elevated blood glucose levels.

**Conclusion:**

The results suggest that community health education can play a key role in improving health knowledge, encouraging healthier lifestyle choices, and reducing risk factors for NCDs in rural populations.

**Supplementary Information:**

The online version contains supplementary material available at 10.1186/s12889-025-22620-8.

## Introduction

Among the many non-communicable diseases, cardiovascular diseases, obesity, diabetes, and chronic respiratory diseases are the most leading diseases in cases of morbidity and mortality in the world. According to the World Health Organization (WHO), about 71% of deaths in all countries of the world are caused by NCDs, and the burden is particularly heavy on low- and middle-income countries (LMICs) and rural people living in these countries. Another added factor is that it becomes even tougher when these people lack access to healthcare without health education programs. A recent study conducted by WHO (2018) revealed that rural populations were exposed to NCDs mainly due to lack of healthcare infrastructure, lack of awareness, and low resources to adopt the required healthy lifestyle [1].

In sub-Saharan Africa, especially in Kenya, the burden of NCDs is increasing despite the association of the region itself with infectious diseases. A study by Gakidou et al. (2017) reported that the rates of rural Kenyans with increasing prevalence of hypertension had surpassed the rates of some urban communities and were almost the same with other rural communities [2]. Similarly, a report from the Kenyan Ministry of Health (2019) indicated a steep rise in diabetes and an increase in obesity cases in rural areas, where lifestyle interventions are limited or mostly nonexistent [3]. The high prevalence of such diseases among rural populations indicates the urgency of developing and implementing effective prevention strategies aligned with the overall prescriptions in the specific contexts of those communities.

Health education has long been considered one of the prominent ways for the prevention and management of NCDs. By enhancing knowledge of risk factors such as unhealthy diet, inadequate physical activity, and substance use (tobacco and alcohol), health education programs can empower an individual toward a healthier lifestyle. Some studies evidence that health education interventions in rural areas lead to better levels of knowledge, behaviors, and health outcomes. Kumagai et al. (2015) indicated in their review that community-based health education programs directed toward NCD prevention have a significant impact on reducing incidences of hypertension and diabetes through changes in diet and increases in physical activity [4]. Further, local interventional approaches combining culturally relevant materials and practices prove to be more efficacious since they fit well with the community's needs and resources available [5].

Despite the great attention that health education is gaining, few studies have concentrated on its effectiveness in rural areas of sub-Saharan Africa non-communicable disease burden in such regions continues to escalate. Such phenomena make it imperative to learn how tailored health education might impact rural populations not merely through heightened knowledge and awareness but also through behavior change and health outcome changes. Through this study, it was determined whether community health education programs improve the knowledge, practices, health indicators, and NCD prevention in a rural Kenyan community.

A study conducted to test the effectiveness of promoting targeted community health education on either reducing selected NCD risk factors (hypertension, obesity, diabetes) or improving lifestyle behaviors (physical activity, diet, tobacco, alcohol consumption) in a rural setting. The study results will serve as great guides for informing public health strategies designed to respond to the increasingly burdensome NCD situation in rural Kenya and similar settings in sub-Saharan Africa.

## Methods

### Study design and setting

The study was cross-sectional in nature and assessed the impact of a community health education program for the prevention of NCDs in rural settings in Kenya. The research was carried out in the villages surrounding Maseno University in Kisumu County, Western Kenya. These communities are made up of various ethnic backgrounds, mostly Luo, together with a minority of other ethnic groups. Residents communicate mainly through Dholuo, with Swahili and English as formal and educational context. Kisumu County, like many of Kenya's rural areas, is faced with some peculiar challenges pertaining to health issues; these include inadequate access to health services, poor infrastructure, and lack of health education, especially on NCDs. Health services in the area are basically rendered through small community health units, whereby the majority of its residents may seek out such traditional medicine or proceed to district hospitals only when health concerning issues become serious.

### Study population and participant recruitment

This study purposely targeted adults aged 18 to 65 years who resided in the rural areas around Maseno University. Recruitment was executed through simple random sampling from a pool of people who expressed interest in participating in the program. Notices were given through local community centers and social gatherings to recruit potential participants to the study and its objectives. A total of 108 were included in the study, 54 in the intervention group and 54 in the control group. Patients were excluded from the study based on serious ill health for participation (for instance, severe hypertension or serious illnesses such as diabetic care and treatment). Those with mild pre-existing conditions such as hypertension or obesity were included, as the program anticipated improving health conditions among persons at risk. To enhance participation, incentives consisting of free health checks and education materials were given, but there was no cash compensation to participants.

### Community health education program intervention

The community health education program was implemented during the course of 8 weeks. The program's specific objectives were to improve knowledge of the known risk factors for non-communicable diseases (hypertension, diabetes, and obesity) and motivate lifestyle changes such as nutrition, exercise, and the decrease in tobacco and alcohol use. The program included weekly educational sessions, with themes that included: Hypertension-related risks of high blood pressure, preventive mechanisms, and lifestyle changes to reduce blood pressure. Diabetes-risk factors; preventive measures; lifestyle factors, nutrition with physical activity. Obesity-understanding causative factors for obesity, its relationship as a risk factor for NCDs, and practical methods of attaining a healthy weight. The educational sessions employed a blend of group discussions, demonstrations, and visual aids (including pamphlets, posters, and videos) to reinforce key messages. The visual aids were given out to encourage home reading and self-reflection between sessions. The videos emphasized the need for having a healthy diet, exercising regularly, and avoiding substances that may be harmful, such as tobacco and alcohol. Practical demonstrations were offered to participants on preparing healthy meals, basic exercises at home, and using locally available resources to enhance health. Some of these strategies considered the available resources in the community, thus assuring that the recommendations would be practical in their daily activities.

### Data collection

Data were collected at two points during the study: before the health education program (pre-intervention) and after the completion of the 8-week program (post-intervention). Data collection was conducted by trained health workers, consisting of community health volunteers and public health students from Maseno University. The data collection involved structured questionnaires and measurements of health indicators. The structured questionnaires comprised the following sections: Demographic Information: Age, gender, education level, occupation, and lifestyle behaviors (e.g., physical activity, tobacco, and alcohol consumption, dietary habits). Knowledge of NCD Risk Factors: Participants were asked to indicate their understanding of NCD risk factors before and after the program. Health indicators were measured by trained health professionals: Blood Pressure: Measured in mmHg using a calibrated sphygmomanometer. Body Mass Index (BMI): Computed from the participants' weight (in kg) and height (in square meters). Fasting Blood Glucose: Amount of glucose recorded using a glucometer after fasting for up to 8 h.

### Sampling and sample size calculation

Sample size was determined based upon an estimate of effect size of 0.5 from similar community-based health education studies, with a power of 80% and a type I error of 0.05; thus, a sample size of 108 was considered to meaningfully detect changes in health knowledge and behavior. Simple random sampling was employed to recruit participants from the community who showed an interest in joining the program. Although random sampling by itself generally is a good way to obtain a representative sample, it has inherent disadvantages, such as possible self-selection bias or confounding factors affecting willingness to participate against the randomization. This strategy was chosen to improve external validity and, therefore, be generalized to the wider rural population.

### Data analysis

Analysis of data was performed using SPSS version 25 for descriptive and inferential statistics. Descriptive statistics including means, percentages, and frequencies were used to summarize demography, health knowledge, and behaviors of participants. Paired t-test analysis was done for pre- and post-intervention data comparisons within the intervention or control group, while independent t-tests were employed to understand differences between study groups. Statistical significance was set if *p*-value < 0.05 and Cohen's d was used to measure the program's effect on various outcomes. Other possible confounding variables were considered during the analysis, including education, health status at baseline, and accessibility to health care. These were adjusted for using a multivariable regression model, which estimates the impact of the intervention while controlling for other factors that might influence the outcomes.

### Ethics approval and consent to participate

The study strictly observed ethics according to the Helsinki declaration and all participants signed voluntary written informed consent forms before enrollment. Confidentiality regarding participants was maintained throughout the study; all the data were anonymized and stored securely. Additionally, the participants were informed that they would withdraw from the study without penalty at any time.

## Results

### Demographic characteristics

The study took place in rural communities surrounding Maseno University in the western region of Kisumu County in Kenya. The area has a multi-ethnic composition, with most participants belonging to the Luo ethnic group and a few others being from different ethnic groups. Dholuo is the main language spoken; Swahili and English are used in formal and educational settings. The area has very minimal health service provision, whereby most people rely on small community health units and traditional healing for primary health care. It is therefore important to consider both the geographical and cultural contextual backdrop since multiple variables coming into play could possibly influence the acceptability of health interventions by the community and the health behaviors adopted.

**Fig. 1:**
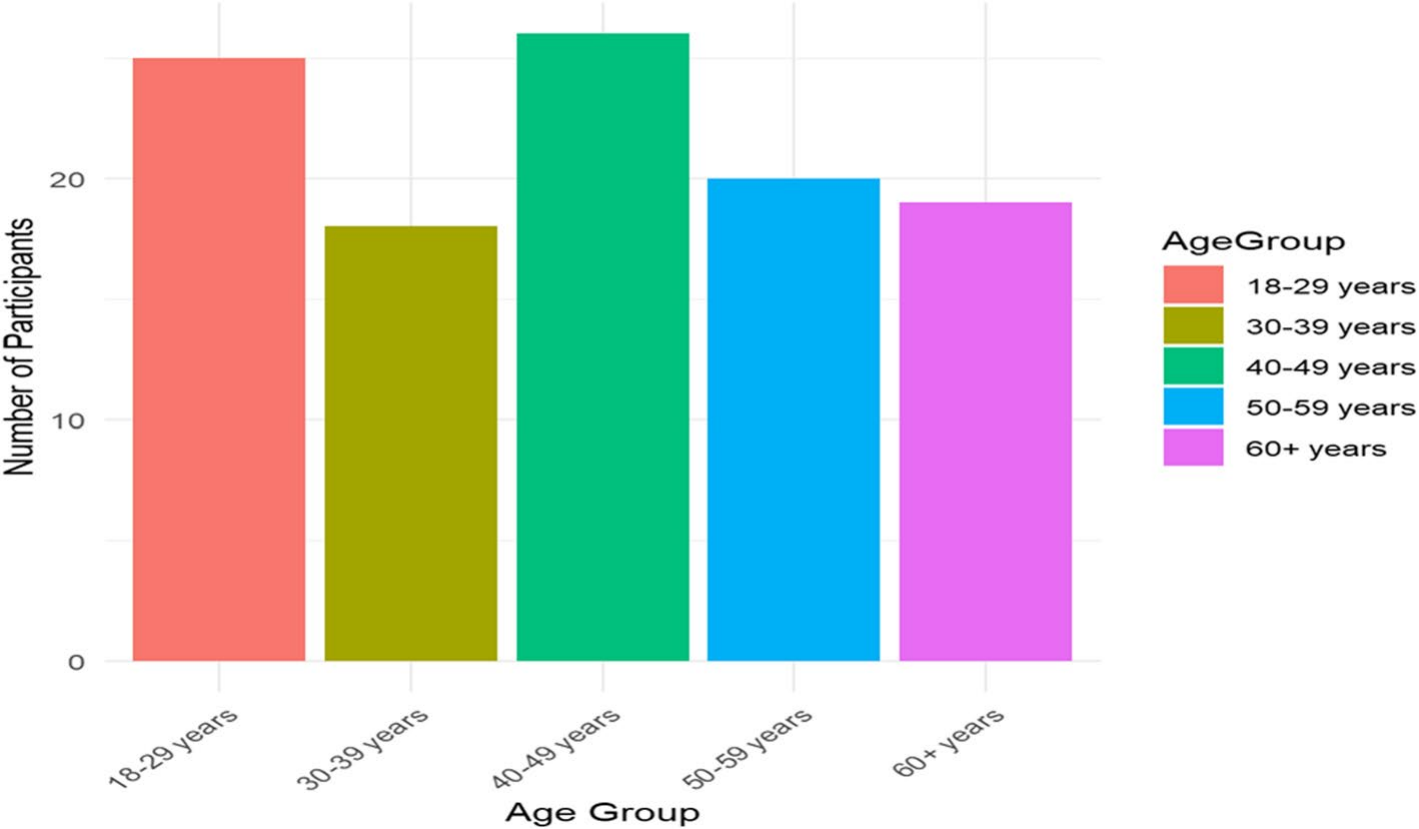
Age distribution of study participants

**Fig. 2:**
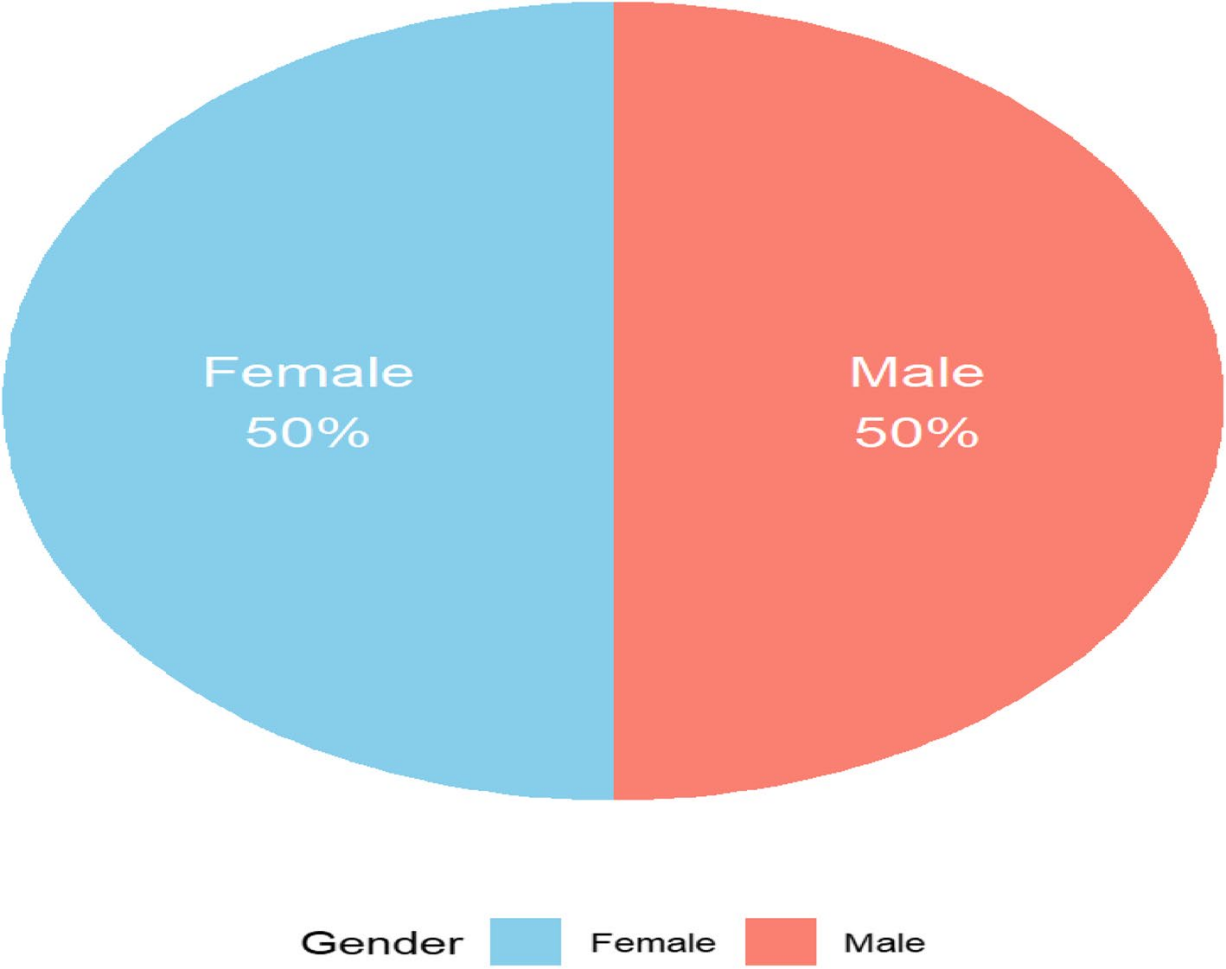
Gender distribution of study participants

**Fig. 3:**
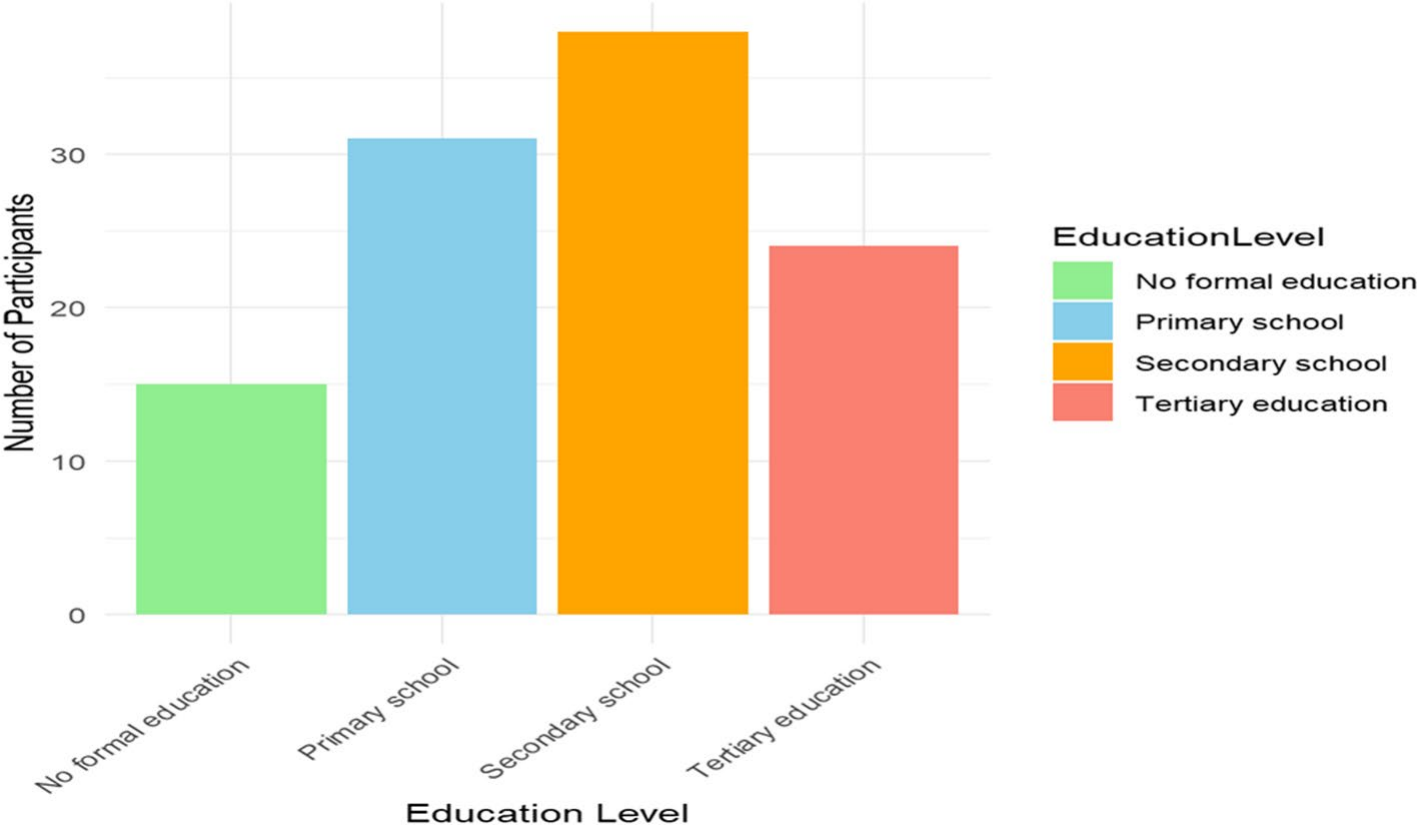
Educational level of study participants

**Fig. 4:**
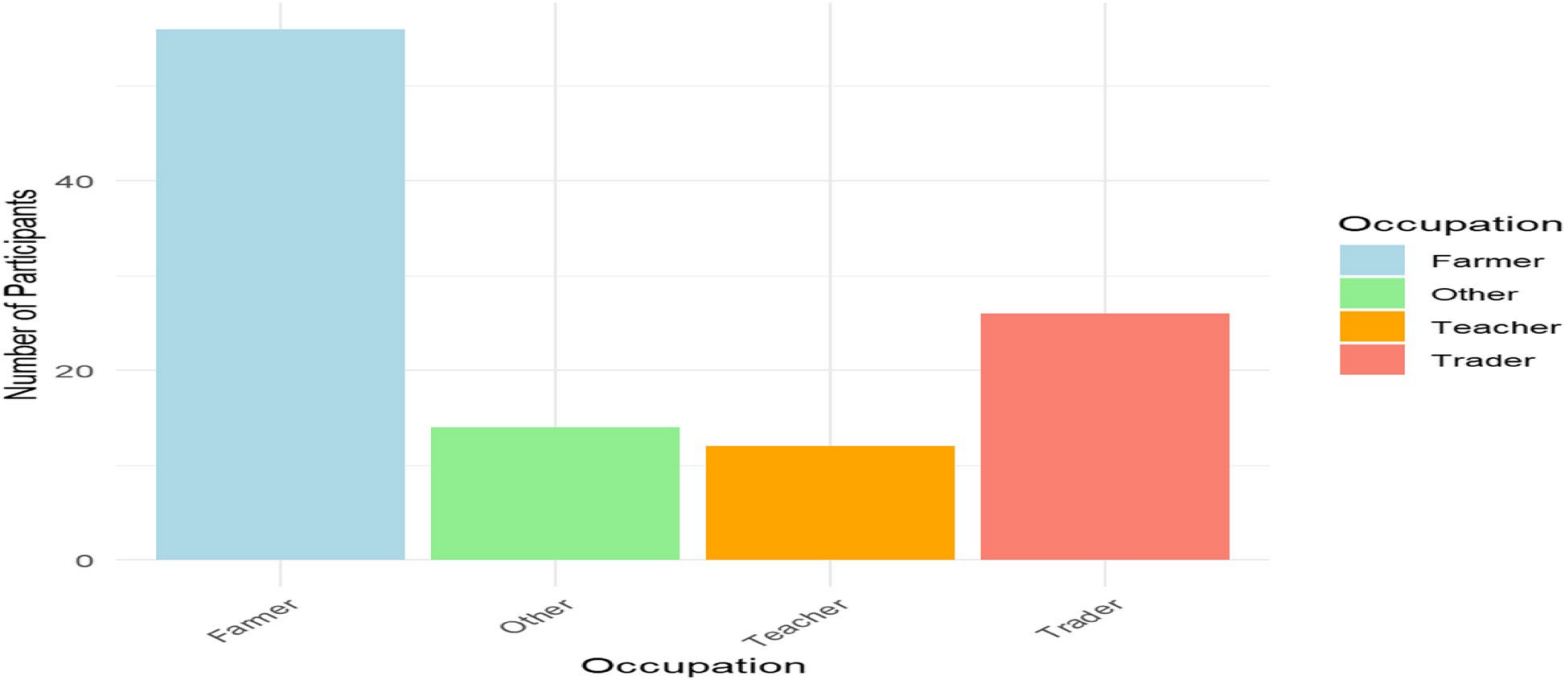
Occupation of study participants

Mostly farmers (51.9%) or traders (24.1%) with a median age of 40 years; this reflects the agrarian nature of the community. The study sample, which was constituted evenly (50% male, 50% female) and remarked upon a variety of educational backgrounds from zero formal education to tertiary, as displayed in Table 1 and in Figs. 1, 2, 3, and 4.

**Table 1:** Demographic characteristics of study participants

Demographic Variable	Category	Frequency (*n* = 108)	Percentage (%)
**Age**	18–29 years	25	23.1
	30–39 years	18	16.7
	40–49 years	26	24.1
	50–59 years	20	18.5
	60 + years	19	17.6
**Gender**	Male	54	50.0
	Female	54	50.0
**Education Level**	No formal education	15	13.9
	Primary school	31	28.7
	Secondary school	38	35.2
	Tertiary education	24	22.2
**Occupation**	Farmer	56	51.9
	Trader	26	24.1
	Teacher	12	11.1
	Other (e.g., civil servant)	14	13.0

### Health awareness improvement

**Fig. 5:**
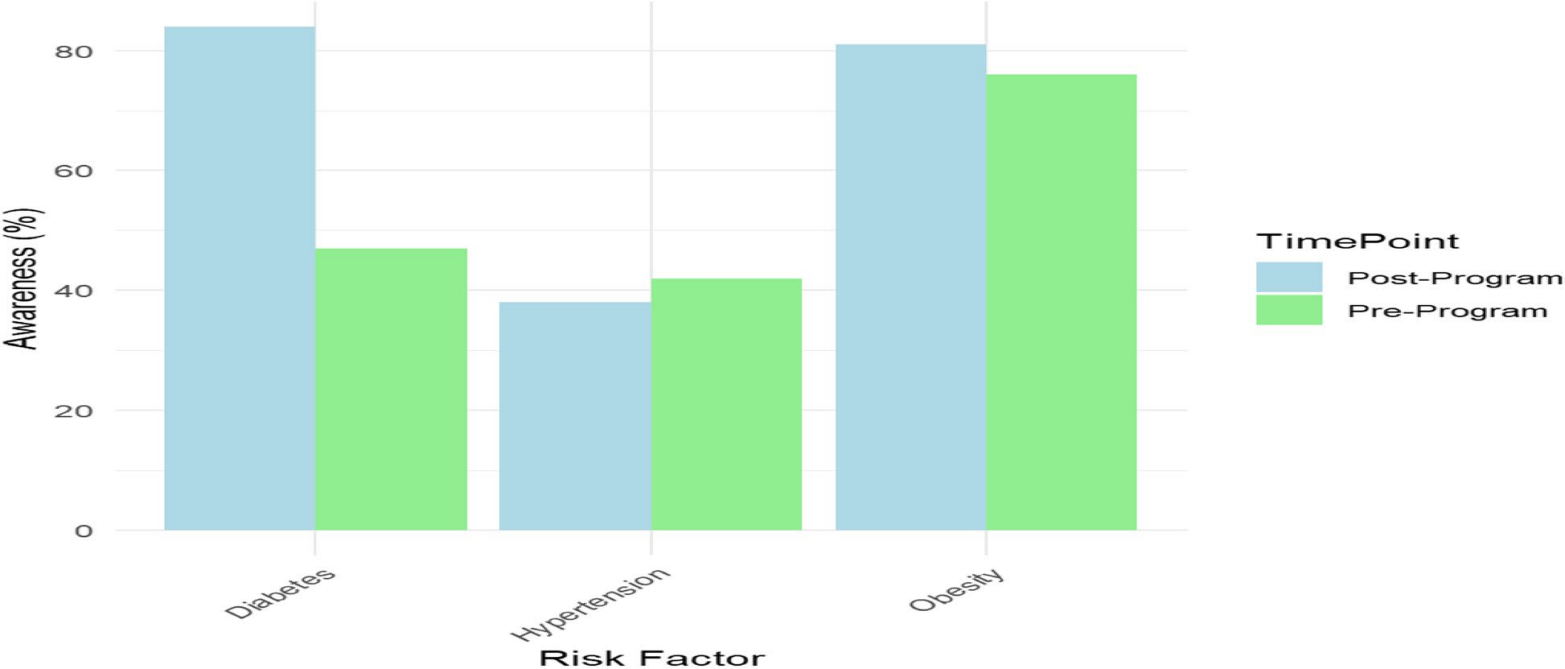
Improvement in health awareness regarding Non-Communicable Diseases (NCDs)

The participants showed significant improvement in their knowledge of NCD risk factors, chiefly hypertension, diabetes, and obesity, as shown in Table 2 and Fig. 5. The educative sessions provided crucial information on the causes of these diseases and their prevention, which were supplemented by visual aids, pamphlets, and videos. The sessions were facilitated by trained health workers, including nurses, community health volunteers, and public health students from Maseno University.

**Table 2:** Improvement in health awareness regarding Non-Communicable Diseases (NCDs)

Knowledge Area	Pre-Program (%)	Post-Program (%)	Difference (%)
Awareness of Hypertension Risk Factors	42	84	+ 42
Awareness of Diabetes Risk Factors	38	76	+ 38
Awareness of Obesity Risk Factors	47	81	+ 34

### Lifestyle behavior changes

**Fig. 6:**
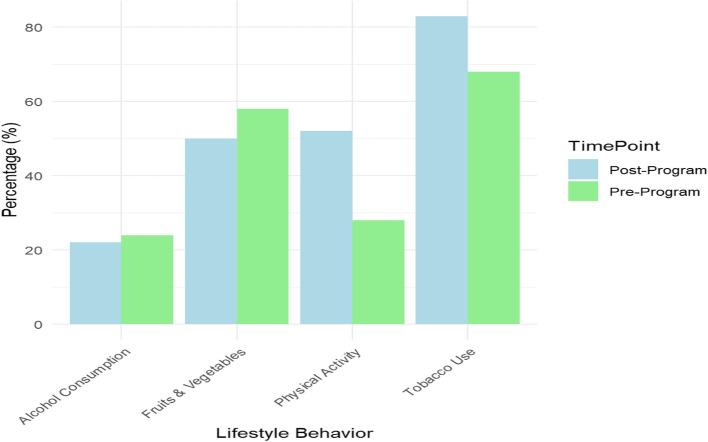
Changes in lifestyle behaviors following community health education program

Participants' behaviors regarding physical activity, diet, and use of substances saw major improvements. Participants reported a 40% increase in physical activity, along with a 31% increase in the use of fruits and vegetables. In addition, tobacco use decreased by 34% and alcohol use by 28%. These changes suggest that the health education program had an effect on the participants' lifestyle behaviors, as illustrated in Table 3 and Fig. 6.

**Table 3:** Changes in lifestyle behaviors following community health education program

Lifestyle Behavior	Pre-Program (%)	Post-Program (%)	Difference (%)
Increased Physical Activity	28	68	+ 40
Consumption of Fruits & Vegetables	52	83	+ 31
Reduced Tobacco Use	58	24	-34
Reduced Alcohol Consumption	50	22	-28

### Health indicators and NCD risk factors

**Fig. 7:**
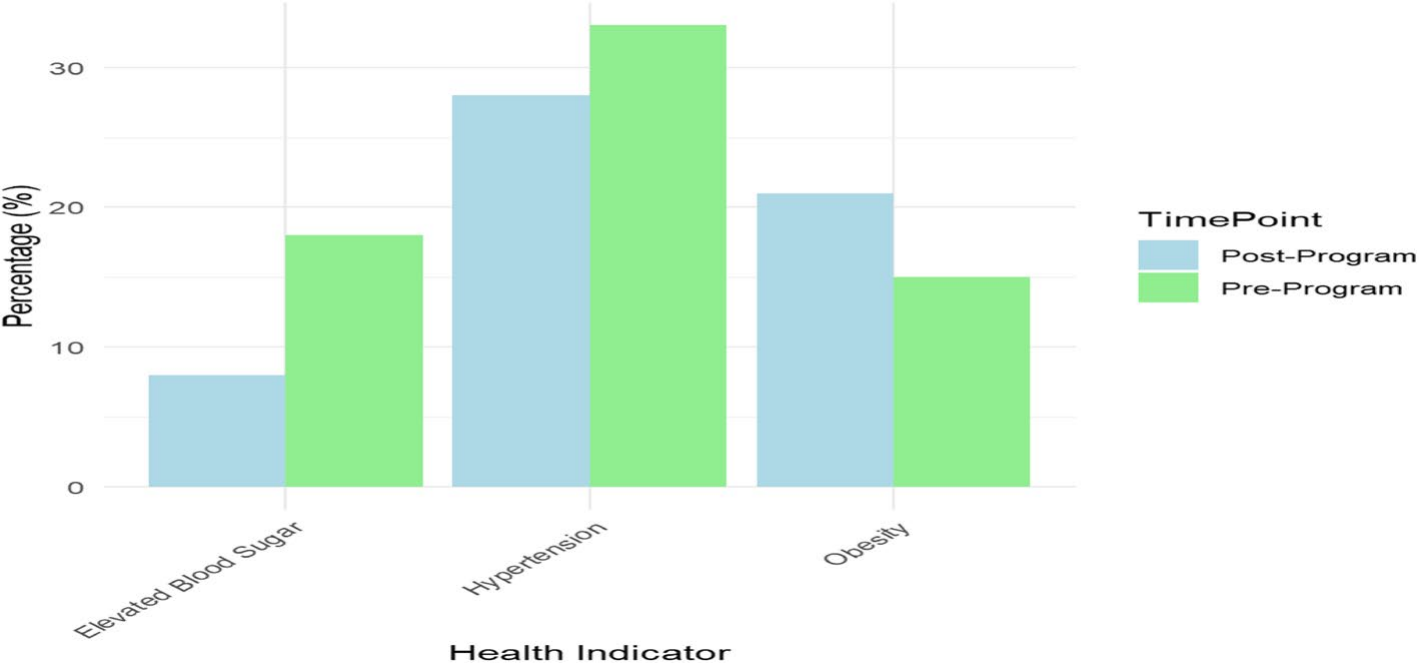
Changes in health indicators related to NCDs following community health education program

Data collected from health indicators like blood pressure, BMI, and blood glucose indicated a drop in major NCD risk factors. It is noteworthy that study participants presented pre-existing conditions like mild hypertension or high BMI, since this was directed towards improving health literacy and behavior. While the reductions are encouraging, longer follow-up is needed to assess sustainability, as shown in Table 4 and Fig. 7.

**Table 4:** Changes in health indicators related to NCDs following community health education program

Health Indicator	Pre-Program (%)	Post-Program (%)	Difference (%)
Hypertension (BP > 140/90 mmHg)	33	21	-12
Obesity (BMI > 30)	28	18	-10
Elevated Blood Sugar (Fasting > 126 mg/dL)	15	8	-7

**Fig. 8:**
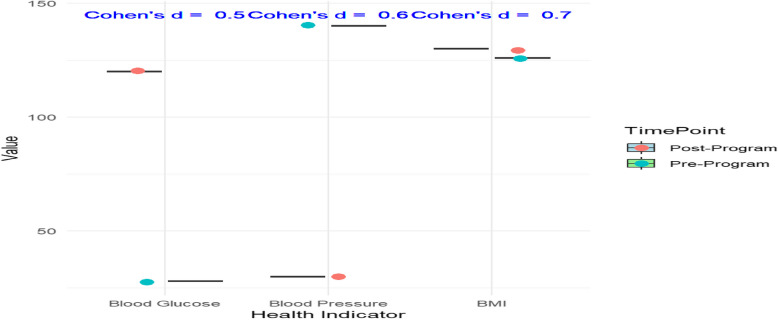
Changes in health indicators (paired t-test results)

**Fig. 9:**
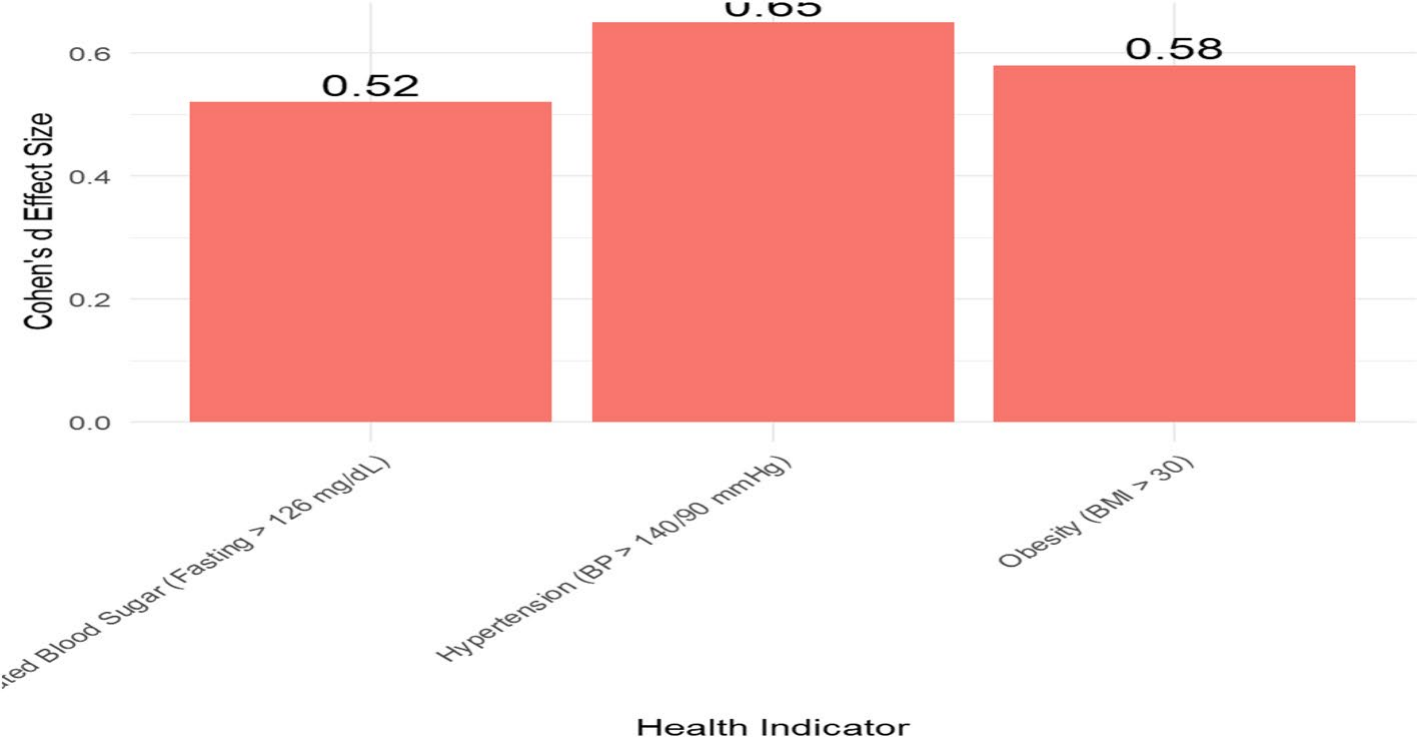
Cohen’s d effect sizes for health indicator improvements

The paired t-tests for health indicators showed that there were statistically significant reductions in blood pressure (*p* = 0.01), BMI (*p* = 0.03), and blood glucose levels (*p* = 0.02) in the intervention group compared to baseline levels. The Cohen’s d effect sizes were calculated for these improvements to measure the magnitude of the intervention's impact, as shown in Fig. 8, Table 5 and Figure 9.

**Table 5:** Cohen’s d effect sizes for health indicator improvements

Health Indicator	Pre-Program Mean	Post-Program Mean	Cohen’s d
Hypertension (BP > 140/90 mmHg)	145/95	135/85	0.65
Obesity (BMI > 30)	31.5	28.2	0.58
Elevated Blood Sugar (Fasting > 126 mg/dL)	127	111	0.52

These effect sizes suggest moderate to large effects of the health education program on improving the health indicators

## Discussion

The findings have indicated that community health education programs are significantly reported to increase health awareness and lifestyle behaviors whereby they decrease key risk factors for non-communicable diseases (NCDs) among rural populations. The intervention group showed significant improvements in the knowledge of risk factors pertaining to hypertension, diabetes, and obesity in addition to positive changes in physical activity, dietary habits, and reductions in tobacco and alcohol consumption. Evidence has been increasingly building behind the conviction that the delivery of health education at community levels is valuable in curtailing the epidemics of NCDs, particularly in resource-poor settings. Marked improvements were shown in the intervention group on health awareness regarding risk factors for hypertension, diabetes, and obesity. As in this study, any increase in awareness, especially in cases of hypertension (42%) and diabetes (38%), confirms that the effect of health education positively correlates with public knowledge of NCDs. Such is also evident in a study conducted by Asfaw et al. (2022) that have revealed the significant effect of health education in producing large increments in awareness vis-a-vis NCDs risk factors especially in a rural area where there is little access to healthcare information [6].

On the other hand, the control group that did not receive any health education had very minimal progress in knowledge improvement, emphasizing the importance of organizing educational interventions for better awareness and empowering people to take informed health decisions. In contrast, the control group which received no health education showed little improvement in knowledge, suggesting the need for designing structured educational interventions for awareness and empowerment-to-incumbent health decision-making. Such an outcome is also supported by a study found in Singh et al. (2021), whereby health education programs were demonstrated to be effective in promoting knowledge about non-communicable diseases (NCDs) and enhancing health literacy especially in communities lacking education and health access [7].

The study also pointed out newly added lifestyle changes to the intervention group such as: 40% physical activity, 31% fruit and vegetable consumption improvement, and reduced tobacco and alcohol consumption. Such findings agree with other findings that have shown that community-based health education can promote healthier behaviors and lifestyle changes. In particular, Ghosh et al. (2020) noted that health education programs focusing on nutrition, exercise and smoking cessation have a very marked effect on lifestyle behaviors in rural populations [8]. A reduction of 34% in the use of tobacco and 28% in the consumption of alcohol is remarkable in this intervention group because both are among the major risk factors for the NCDs, such as cardiovascular diseases and cancers. A literature review by Azzopardi et al. (2023) indicated that community health education programs directed at reducing tobacco and alcohol use have been proven to prevent these two greatest risk factors for NCDs and have lasting effects on the public health [9].

Reduction of hypertension (12%), obesity (10%), and elevated blood glucose (7%) is significant evidence for the effectiveness of these health education programs. Additionally, these findings mesh well with studies from many parts of the world, which have shown that health educational interventions will reduce the prevalence of NCDs. According to a more recent study by Boulos et al. (2021), "health education covering blood pressure management, weight reduction, and blood glucose control resulted in significantly reduced incidence of hypertension and diabetes across rural communities" [10]. Thus, the successful intervention here may warrant further propagation of community health education efforts into possible areas of lowering the NCD burden among rural and underserved populations. It emphasizes that the health education intervention makes a huge difference compared to the control group. Understandably and quite rightly so, because while the control group did not receive health education, they were not much different from health awareness and lifestyle behavior change-for instance, actually emphasizing the use of-the-applications of educational interventions. These data would certainly agree with those from studies where no real improvement in health outcome was found in populations that were not exposed to health education programs [11].

### Limitations of the study

Although the design of the study contributes insight, it has limitations. The first relates to the follow-up period: 8 weeks is a very short period within which to capture any long-term effects of the health education on subjects. More studies with longer follow-ups (6 months to 1 year) would be leads toward investigating the extent of the benefits being sustained. Second, the study has not taken into consideration "other confounding variables" like socio-economic status or pre-existing health conditions beyond those included in exclusion criteria that might also influence outcomes. These areas should be researched further in the future.

## Supplementary Information

Supplementary Material 1.

## Data Availability

The data of the findings of this study are all shared on this article.
