# Supplementary material for: Assessing the impact of community health education programs on preventing non-communicable diseases in rural areas
Source: BMC Public Health. 2025 Nov 27;25:4176. doi: 10.1186/s12889-025-22620-8 (PMC12659474; doi:10.1186/s12889-025-22620-8)
Supplement: Supplementary file 1 — Supplementary Material 1. [file 12889_2025_22620_MOESM1_ESM.pdf]

Questionnaire: Impact of Community Health Education Programs on Preventing Non-Communicable Diseases

This questionnaire is designed to assess the impact of community health education programs on preventing non-communicable diseases (NCDs) in rural populations. NCDs, such as hypertension, diabetes, obesity, and other chronic diseases, have become significant health concerns in many rural areas where access to healthcare services and health education may be limited. The goal of this study is to evaluate how health education programs can improve knowledge, lifestyle behaviors, and health outcomes related to NCD prevention.

Your responses to the questions will help researchers understand how health education influences awareness and behavioral changes in relation to NCDs. All information provided will be treated with confidentiality, and your participation is voluntary. By participating, you will contribute to the improvement of public health strategies aimed at reducing the burden of NCDs in rural communities.

Section 1: Demographic Information

1. **Age:**
   ☐ 18-29 years
   ☐ 30-39 years
   ☐ 40-49 years
   ☐ 50-59 years
   ☐ 60 years and above
2. **Gender:**
   ☐ Male
   ☐ Female
3. **Highest level of education completed:**
   ☐ No formal education
   ☐ Primary school
   ☐ Secondary school
   ☐ Tertiary education (College/University)
4. **Occupation:**
   ☐ Farmer
   ☐ Trader
   ☐ Teacher
   ☐ Civil servant
   ☐ Other (Please specify): ______________________
5. **Do you live in a rural or urban area?**
   ☐ Rural
   ☐ Urban

Section 2: Health Knowledge (NCD Risk Factors)

Please indicate your level of knowledge on the following topics before and after the community health education program.

1. **Hypertension (High Blood Pressure):**
   Before the program:
   ☐ Not aware of the risk factors
   ☐ Aware of some risk factors
   ☐ Fully aware of all risk factors

After the program:
☐ Not aware of the risk factors
☐ Aware of some risk factors
☐ Fully aware of all risk factors

2. **Diabetes:**
   Before the program:
   ☐ Not aware of the risk factors
   ☐ Aware of some risk factors
   ☐ Fully aware of all risk factors

After the program:
☐ Not aware of the risk factors
☐ Aware of some risk factors
☐ Fully aware of all risk factors

3. **Obesity (Overweight):**
   Before the program:
   ☐ Not aware of the risk factors
   ☐ Aware of some risk factors
   ☐ Fully aware of all risk factors

After the program:
☐ Not aware of the risk factors
☐ Aware of some risk factors
☐ Fully aware of all risk factors

4. **Other NCDs (Stroke, Cancer, etc.):**
   Before the program:
   ☐ Not aware of the risk factors
   ☐ Aware of some risk factors
   ☐ Fully aware of all risk factors

After the program:
☐ Not aware of the risk factors

☐ Aware of some risk factors
☐ Fully aware of all risk factors

Section 3: Lifestyle Behaviors

Please indicate the frequency of the following behaviors before and after the community health education program.

1. **Physical Activity (e.g., walking, running, exercise):**
   Before the program:
   ☐ None
   ☐ Rarely (1-2 times/week)
   ☐ Occasionally (3-4 times/week)
   ☐ Regularly (5 or more times/week)

After the program:
☐ None
☐ Rarely (1-2 times/week)
☐ Occasionally (3-4 times/week)
☐ Regularly (5 or more times/week)

2. **Dietary Habits (Consumption of fruits and vegetables):**
   Before the program:
   ☐ Rarely
   ☐ Occasionally
   ☐ Frequently
   ☐ Always

After the program:
☐ Rarely
☐ Occasionally
☐ Frequently
☐ Always

3. **Tobacco Use (Cigarettes, smokeless tobacco, etc.):**
   Before the program:
   ☐ Never
   ☐ Occasionally
   ☐ Regularly
   ☐ I have quit tobacco use

After the program:
☐ Never
☐ Occasionally

☐ Regularly
☐ I have quit tobacco use

4. **Alcohol Consumption:**
   Before the program:
   ☐ Never
   ☐ Occasionally
   ☐ Regularly
   ☐ Frequently

After the program:
☐ Never
☐ Occasionally
☐ Regularly
☐ Frequently

5. **Stress Management (e.g., relaxation techniques, counseling, etc.):**
   Before the program:
   ☐ Never
   ☐ Occasionally
   ☐ Regularly
   ☐ Always

After the program:
☐ Never
☐ Occasionally
☐ Regularly
☐ Always

Section 4: Health Indicators (Self-Reported Health Status)

Please indicate your health status before and after the program.

1. **Blood Pressure (BP):**
   Before the program:
   ☐ Normal (≤ 120/80 mmHg)
   ☐ Elevated (120-139/80-89 mmHg)
   ☐ Hypertension (≥ 140/90 mmHg)
   ☐ I don’t know

After the program:
☐ Normal (≤ 120/80 mmHg)
☐ Elevated (120-139/80-89 mmHg)

☐ Hypertension (≥ 140/90 mmHg)
☐ I don't know

2. **Body Mass Index (BMI):**
   Before the program:
   ☐ Normal weight (18.5-24.9 kg/m²)
   ☐ Overweight (25-29.9 kg/m²)
   ☐ Obese (≥ 30 kg/m²)
   ☐ I don't know

After the program:
☐ Normal weight (18.5-24.9 kg/m²)
☐ Overweight (25-29.9 kg/m²)
☐ Obese (≥ 30 kg/m²)
☐ I don't know

3. **Fasting Blood Glucose (Blood sugar levels):**
   Before the program:
   ☐ Normal (≤ 100 mg/dL)
   ☐ Elevated (100-125 mg/dL)
   ☐ Diabetic (≥ 126 mg/dL)
   ☐ I don't know

After the program:
☐ Normal (≤ 100 mg/dL)
☐ Elevated (100-125 mg/dL)
☐ Diabetic (≥ 126 mg/dL)
☐ I don't know

Section 5: General Feedback

1. **How would you rate the health education program in terms of its usefulness?**
   ☐ Very Useful
   ☐ Useful
   ☐ Neutral
   ☐ Not Useful

2. **Which topic did you find most helpful?**
   ☐ Hypertension
   ☐ Diabetes
   ☐ Obesity
   ☐ Stress Management
   ☐ Diet and Nutrition

☐ Physical Activity
☐ Tobacco and Alcohol Use

3. **Do you feel more confident in managing your health after the program?**
☐ Yes, a lot more confident
☐ Yes, somewhat confident
☐ No change
☐ No, less confident

4. **Would you recommend this health education program to others in your community?**
☐ Yes
☐ No

Thank you for your responses. End of Questionnaire
